# Defective oxytocin function: a clue to understanding the cause of autism?

**DOI:** 10.1186/1741-7015-7-63

**Published:** 2009-10-22

**Authors:** Fiorella Gurrieri, Giovanni Neri

**Affiliations:** 1 Institute of Medical Genetics, Catholic University School of Medicine, Rome, Italy

## Abstract

The autism spectrum disorders are a group of conditions with neurobehavioral impairment affecting approximately 0.6% of children. The clinical presentation is complex and the etiology is largely unknown, although a major role of genetic factors is widely accepted. A number of genetic studies led to the identification of genes and/or copy number variants whose alterations are associated with autism, but no specific factor has been found so far to be responsible for a substantial proportion of cases. Epigenetic modifications may also play a role, as demonstrated by the occurrence of autism in genetic conditions caused by mutations in imprinted genes or regions.

The article by Gregory *et al*. published this month in *BMC Medicine*, reports on genomic and epigenetic alterations of *OXTR*, the gene encoding the receptor for oxytocin. The involvement of this gene was suggested by its deletion in an autistic patient. The subsequent analysis of a group of unrelated autistic subjects did not show an *OXTR *deletion, but rather hypermethylation of the gene promoter, with a reduced mRNA expression.

These findings address two major points of the current debate on the etiology and pathogenesis of autism: the role of oxytocin, known to be involved in modeling human behavior, and the possible involvement of epigenetic mechanisms. The nature of this epigenetic dysregulation is unknown but, if proved to be true, might explain the failure to identify sequence alterations in a host of candidate genes. Practical implications of these findings may be forthcoming, however not before extension and validation on a larger scale have confirmed their value.

See the associated research paper by Gregory et al:

## Background

This month in *BMC Medicine*, Gregory *et al*. [1] report on a potential new clue to understanding the etiology of autism.

The autism spectrum disorders (ASDs) are a group of neurodevelopmental conditions characterized by impairment in social, emotional and communicative skills and by stereotyped motor and mental processes with an onset in the first 3 years of life. ASD affects approximately 0.6% of children[2]. The number of children diagnosed with this condition has greatly increased in recent decades, likely due to increased awareness, reduced stringency of diagnostic criteria and, possibly, other factors of unknown nature [3].

Regarding the etiology, it is generally accepted that genetic factors play a major role and it has become clear that, with the exception of a minority of instances (about 1%) in which the phenotype is caused by a single gene alteration, the genetic component leading to autism is complex, being based on interactions of multiple genetic changes and/or epigenetic regulation of gene expression.

Because of this complexity, decades of experimental work, even with the aid of sophisticated molecular genetic tools (such as high resolution total genome quantitative analysis, array expression analysis or high resolution single nucleotide polymorphism (SNP) array platforms) have failed to identify specific causes in any substantial proportion of cases [4,5]. Nonetheless, quantitative whole-genome analyses promise to identify one or a few autism-specific copy number variants (CNVs) harboring genes whose dosage alterations could be critical in causing ASD. From a number of array comparative genomic hybridization (array CGH) studies [6], even on large cohorts of patients, we have learnt that up to 10% of sporadic ASD cases do show *de novo *CNVs, some non-randomly associated with ASD, although none of these have been so far proven to harbor major genes causing or predisposing to ASD.

Likewise, several biological parameters, such as the postsynaptic receptor density [7,8], the intercellular adhesion mechanisms [9,10], the immune system [11-16], some hormonal exposures [17-22], imbalances in specific neurotransmitters [23,24] and the epigenetic regulation of gene expression [25,26] have emerged as possibly involved in the pathogenesis of ASD.

### *OXTR *in ASD

This month in *BMC Medicine*, Gregory *et al*. show that there is genomic and epigenetic evidence of a reduced function of the oxytocin receptor in autism. They identified a genomic deletion containing the gene encoding for the oxytocin receptor (*OXTR*) in an autistic male individual and in his mother, who probably experienced obsessive-compulsive disorder (OCD). The equally affected brother did not have the same deletion but rather a hypermethylation of specific CpG islands likely to cause a reduced expression of the *OXTR *gene. It would be interesting to know the methylation status of the *OXTR *allele also in the deleted proband and in the mother, and to measure its level of expression. Likewise, one would want to know whether the maternal deletion was *de novo *or inherited. Could the *OXTR *gene be imprinted? So far there is no evidence of a possible monoallelic, parental-specific expression in any tissue.

Regardless, the above findings prompted the analysis of the methylation status of the *OXTR *gene in a number of samples from autistic subjects, including brain tissue from the temporal cortex as well as peripheral blood lymphocytes. Although the sample is not a large one, the authors found a hypermethylation of specific CpG islands and a correspondingly reduced expression of the *OXTR *gene. This brings up the question of how did this hypermethylation occur? Was it environmentally induced or genetically determined?

The findings of Gregory *et al*. address two major points in the current debate on the cause and pathogenesis of autism: one related to the role of oxytocin and its receptor, the other to the possible role of epigenetic mechanisms.

Regarding the former, there is growing evidence that the oxytocin-vasopressin pathway has an influence on social behavior both under normal circumstances and in a clinical setting [19,20,27,28]. It has been reported that mice lacking the oxytocin gene show social deficits [29], thus strengthening the role of this nonapeptyde in modeling individual behavior. In addition, association studies in autistic cohorts have shown linkage with polymorphisms in the vasopressin and *OXTR *genes [21,30-32]. Duplications of the 3p25-3p26 genomic region, leading to overexpression of the *OXTR *gene, have been associated with a pervasive developmental disorder in a patient with obesity and behavioral issues [33], thus suggesting that increased *OXTR *expression can cause behavioral phenotype falling within the ASD spectrum.

### Epigenetic modification of *OXTR*

The second issue raised by Gregory *et al*. deals with the epigenetic inhibition of *OXTR *expression in ASD. Such epigenetic modification, at least as reported so far, does not seem to be sequence based but rather of a different, as yet unknown nature. This might explain why researchers have been looking for decades for genetic mutations in ASD and yet have found almost none. An epigenetic mechanism would justify the 'unusual', non-Mendelian familial aggregations of ASD. In this respect, even the family with *OXTR *deficiency reported by Gregory *et al*. shows an unusual genotype-phenotype correlation, in that the same phenotype is caused by alterations of the same gene but due to different molecular defects (deletion versus hypermethylation).

Also, the possibility that in most ASD patients there might be an epigenomic instability is of interest in consideration of the fact that it has been shown that the epigenetic status in early fetal development can be reprogrammed by maternal behavior in a reversible way [34]. Therefore, other environmental factors, yet to be discovered, might also be able to reprogram the epigenotype of the embryo.

The epigenetic nature of ASD has been already suspected based on many instances, such as the association of ASD with conditions caused by mutations of imprinted genes like Angelman, Fragile X or Rett syndromes, or with quantitative alterations in regions like the 15q11-q13, also known to be imprinted [25].

## Conclusion

The findings by Gregory *et al*. seem to open new avenues in the research on ASD, one involving the oxytocin pathway, the other the human methylome. Thus, future research in the field may take sharp turns, searching for genetic alterations responsible for ASD beyond DNA sequencing and into epigenetic regulation of gene function. Of course, before these findings can be applied as diagnostic tools or biomarkers of ASD, or can even lay down a basis for therapeutic approaches, it is necessary to confirm them in larger cohorts of patients and controls. It is true that in ASD replication studies have often failed, although this should not stop us from striving to solve this puzzle. Time and further studies will tell us whether the results seen here live up to their initial promise.

## Competing interests

The authors declare that they have no competing interests.

## Authors' contributions

Both authors contributed equally to the writing of this article.

## Pre-publication history

The pre-publication history for this paper can be accessed here:


